# Scalable 3D Printed Molds for Human Tissue Engineered Skeletal Muscle

**DOI:** 10.3389/fbioe.2019.00020

**Published:** 2019-02-14

**Authors:** Andrew J. Capel, Rowan P. Rimington, Jacob W. Fleming, Darren J. Player, Luke A. Baker, Mark C. Turner, Julia M. Jones, Neil R. W. Martin, Richard A. Ferguson, Vivek C. Mudera, Mark P. Lewis

**Affiliations:** ^1^School of Sport, Exercise and Health Sciences, Loughborough University, Loughborough, United Kingdom; ^2^Institute of Orthopaedics and Musculoskeletal Sciences, RNOH, University College London, London, United Kingdom; ^3^University Hospitals of Leicester NHS Trust, Leicester, United Kingdom

**Keywords:** primary skeletal muscle, tissue engineering, 3D printing, skeletal muscle physiology, bioengineering

## Abstract

Tissue engineered skeletal muscle allows investigation of the cellular and molecular mechanisms that regulate skeletal muscle pathology. The fabricated model must resemble characteristics of *in vivo* tissue and incorporate cost-effective and high content primary human tissue. Current models are limited by low throughput due to the complexities associated with recruiting tissue donors, donor specific variations, as well as cellular senescence associated with passaging. This research presents a method using fused deposition modeling (FDM) and laser sintering (LS) 3D printing to generate reproducible and scalable tissue engineered primary human muscle, possessing aligned mature myotubes reminiscent of *in vivo* tissue. Many existing models are bespoke causing variability when translated between laboratories. To this end, a scalable model has been developed (25–500 μL construct volumes) allowing fabrication of mature primary human skeletal muscle. This research provides a strategy to overcome limited biopsy cell numbers, enabling high throughput screening of functional human tissue.

## Introduction

Physiologically representative models of skeletal muscle development, regeneration and adaptation will underpin the next generation of understanding regarding the pathophysiological characteristics regarding health and disease in this tissue. However, investigating the cellular and molecular mechanisms that regulate muscle function *in vivo* is problematic, with clear experimental limitations associated with both *in vivo* human and animal models (Friedmann-Bette et al., 2012). As such, establishing a highly biomimetic model that accurately represents the native *in vivo* function is of paramount importance. Tissue engineering (TE) offers an alternative experimental platform to investigate skeletal muscle development and post-natal adaptation and function. However, many current models are not amenable to incorporation of primary human tissue, which are often limited in experimental throughput due to the complexities associated with recruiting tissue donors, donor specific variations, as well as cellular senescence associated with continued passaging. Therefore, a model that is reproducible when scaling down cell number is fundamental in generating high-powered experiments using primary human derived cells. In many current TE skeletal muscle hydrogels, a single human microbiopsy would supply viable cells that generate ~10 constructs. The same number of cells could, however, be used to generate over 50 times this number of constructs. Such increases would represent a significant step forward when using primary human tissue as a cell source for the study of muscle physiology and disease in TE models.

*In vivo* skeletal muscle comprises bundles of highly aligned and differentiated post-mitotic multinucleated fibers, which are organized in a hierarchical manner within an extracellular matrix (ECM). When seeking to develop a TE model that is biomimetic, it must support alignment and differentiation of seeded muscle precursor cells (MPC's) into multinucleated myotubes in three-dimensions, whilst retaining the capacity to generate the force required to perform functional contractions, representative of those seen *in vivo* (Liao and Zhou, 2009). A number of TE models have been proposed using synthetic and naturally derived polymers. Synthetic scaffolds and molds often elicit cellular alignment by providing topographical signals depending on the particular manufacturing method utilized; including electrospinning (Aviss et al., 2010) and micro-patterning (Zatti et al., 2012) of substrates. Despite this, these scaffolds fail to replicate the three-dimensional structure of *in vivo* tissue (Bian and Bursac, 2008) and are often too stiff to facilitate MPC differentiation (Engler et al., 2004). Therefore, naturally derived polymers (most commonly collagen and fibrinogen) that can be manipulated to develop three-dimensional structures are considered more favorable due to their capacity to facilitate tissue stiffness that physiologically resembles *in vivo* skeletal muscle (Vandenburgh et al., 1988; Cheema et al., 2003; Brady et al., 2008; Boonen et al., 2010; Vandenburgh, 2010; Hinds et al., 2011; Langelaan et al., 2011; Truskey et al., 2013; Juhas et al., 2014; Madden et al., 2015).

Models whereby skeletal muscle collagen based constructs are engineered to stimulate the alignment and fusion of seeded MPC's along a single axis, have previously been reported using cell sources from primary rodent (Hinds et al., 2011; Smith et al., 2012), primary human (Powell et al., 2002; Mudera et al., 2010; Martin et al., 2013; Madden et al., 2015), and cell lines (Cheema et al., 2003; Gawlitta et al., 2007). Using these cells, longitudinally orientated parallel arrays of myotubes are evident that closely represent the *in vivo* fascicular structure (Powell et al., 2002; Cheema et al., 2003; Gawlitta et al., 2007; Hinds et al., 2011; Sharples et al., 2012; Smith et al., 2012; Player et al., 2014). These cell laden constructs are set between two fixed points in a mold, which provides resistance to cell mediated contraction of the collagen matrix, contraction of the matrix may be accompanied by a level of collagen degradation reducing the size of the constructs, although this does not affect the integrity of the constructs (Grover et al., 2012). Predictable lines of longitudinal strain are generated as the cells begin to attach (Eastwood et al., 1996, 1998a,b) providing the mechanical stimuli required to promote cell alignment and fusion. As a consequence, it is possible to develop tissue that represents the morphological structure of *in vivo* skeletal muscle (Smith et al., 2012). Despite the utility of the reported models, there remains a need to generate scaled models that can produce human skeletal muscle constructs with reduced cell number requirements to maximize experimental output from primary donor biopsies. Furthermore, the molds previously utilized are typically highly bespoke in nature, resulting in difficulties when reproducing such models and therefore limiting the wide-ranging applicability of these systems. Here, the creation of mold designs that are freely available, and are accurately and instantaneously reproducible are required.

Scaffolds that support biology have been manufactured through a number of microfabrication processes including chemical etching, photolithography, soft lithography, and micromachining (Faustino et al., 2016). Many of these microfabrication techniques involve fabricating a master or template, typically using polydimethylsiloxane (PDMS) as the molding material. There are many desirable properties that make PDMS an ideal material for biological applications, including its low cost, optical transparency, high gas permeability and biocompatibility. However, it is known to chemically swell in a number of solvents and will absorb many hydrophobic compounds. These microfabrication techniques also require the master to be chemically bonded to a second surface, a step that often causes misalignment and poor interlaminar adhesion. This results in labor intensive processing, requiring specialist equipment and training, as well as the provision of clean room facilities. Consequently, design complexity increases manufacturing costs, making prototyping cost and time inefficient (Capel et al., 2018). Recent advances in three-dimensional (3D) printing technologies have allowed the manufacture of biocompatible scaffolds that support cell and tissue growth (An et al., 2015). 3D printing or additive manufacturing is the established terminology describing a range of manufacturing processes that can produce parts with complex and highly customisable 3D geometries (Masood, 1996; Mazzoli, 2013; Skoog et al., 2014; Zhang and Zhang, 2015). In 3D printing, parts are built layer-by-layer, using processes, such as material extrusion, material jetting, vat photo-polymerisation, sheet lamination, powder bed fusion, binder jetting and direct energy deposition (Capel et al., 2013). Utilizing digitally driven printing processes allows for the custom design and prototyping of molds with attachments that are specific to particular tissue types, prior to production of a final functional mold or scaffold. The rapid and relatively inexpensive nature of 3D printing also makes such a method suitable for laboratories to produce molds “in-house,” rather than have to commission production from commercial manufacturing companies.

Building on previous work in our laboratory using large collagen hydrogels [5 and 3 mL volumes (Brady et al., 2008; Smith et al., 2012)], the present research proposes the use of two well-established printing processes, fused deposition modeling (FDM) and laser sintering (LS) to manufacture scalable, reproducible and cost-effective molds. Both of these processes have commercially available benchtop systems, capable of manufacturing functional parts in a range of biocompatible materials (Rimington et al., 2017, 2018). For this research, FDM parts were printed in polylactic acid (PLA), whilst LS parts were printed in polyamide-12 (PA-12). Both of these materials are commercially available and suitable printing parameters are well established amongst the literature (Capel et al., 2013; Rimington et al., 2017). PA-12 is autoclavable making parts reusable for culture applications, whilst the material cost of printing these inserts in PLA (via FDM) is negligible (<£0.05 per insert) so they can be disposed of after a single use. Here we present a rapid, reliable and scalable method for fabricating TE primary skeletal muscle using 3D printed molds. Furthermore, the method reported supports the generation of multiple construct arrays of human derived tissue via scaled reductions in cell density. Such constructs are capable of representing native *in vivo* tissue structure and function. Crucially, the freely available designs for commercially available instrumentation utilized in this work will facilitate the accessible and cost-effective reproduction of TE human skeletal muscle *in vitro*. All designs are available to download at the following URL: https://figshare.com/projects/3D_Printed_Tissue_Engineering_Scaffolds/36494.

## Results

### 3D Printed Inserts Elicit Reproducible Extracellular Matrix Deformation in Tissue Engineered Murine Skeletal Muscle

**Figure 1:**
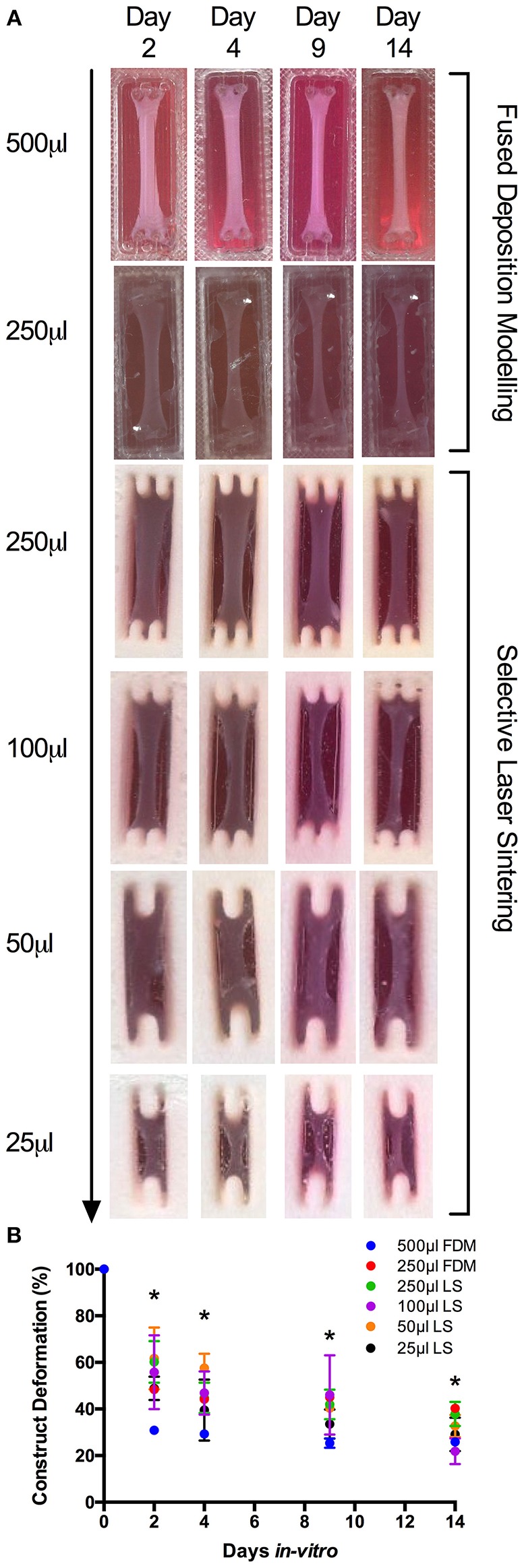
**(A)** Macroscopic images of fused deposition modeling (FDM, 500–250 μL) and laser sintering (LS, 250–25 μL) skeletal muscle constructs over 2, 4, 9, and 14 days culture. **(B)** Construct deformation analysis of entire scaled range (500–25 μL) after 2, 4, 9, and 14 days culture. Data presented as mean ± SD from *n* ≥ 3 experimental repeats in each condition. ^*^P ≤ 0.05. For specific mold dimensions see Table S1.

Measuring construct width provides an indication of cell attachment and remodeling and as such, images of the macroscopic contraction of the constructs were taken throughput the culture period (Smith et al., 2012). Macroscopic analysis of scaled constructs (Figure S1) outlined consistent remodeling of the extracellular matrix within a scaled range of 500–25 μL after 14 days in culture. Analysis of FDM 500 μL constructs (https://doi.org/10.17028/rd.lboro.6969851.v1) demonstrated potentiated early matrix remodeling after 2 days; compared to LS 250, 100 (https://doi.org/10.17028/rd.lboro.6969806.v1) and 50 μL (https://doi.org/10.17028/rd.lboro.6969797.v1) constructs (*P* = 0.015, *P* = 0.049, *P* = 0.010), 4 days culture compared to LS 50 μL (*P* = 0.003) and 9 days compared to LS 100 μL (*P* ≤ 0.05, Figure 1). However, all LS collagen gels displayed comparable remodeling to that of the FDM 500 μL after 14 days in culture, despite initial reductions in deformation observed in LS constructs (250–50 μL) across a range of time-points. Although homogenous macroscopic reductions in 250 μL gel surface areas were observed over time between FDM and LS processes (https://doi.org/10.17028/rd.lboro.6969848.v1), significantly greater matrix remodeling was evident between FDM 500 and 250 μL hydrogels after 14 days in culture (*P* = 0.017). This suggested a preferential detachment of the construct matrix from the printed molds in the larger gel volumes, facilitating an increased rate of construct deformation.

### 3D Printed Inserts Promote Reproducible Morphology Within Tissue Engineered C_2_C_12_ Constructs Irrespective of Printing Process or Construct Volume

**Figure 2:**
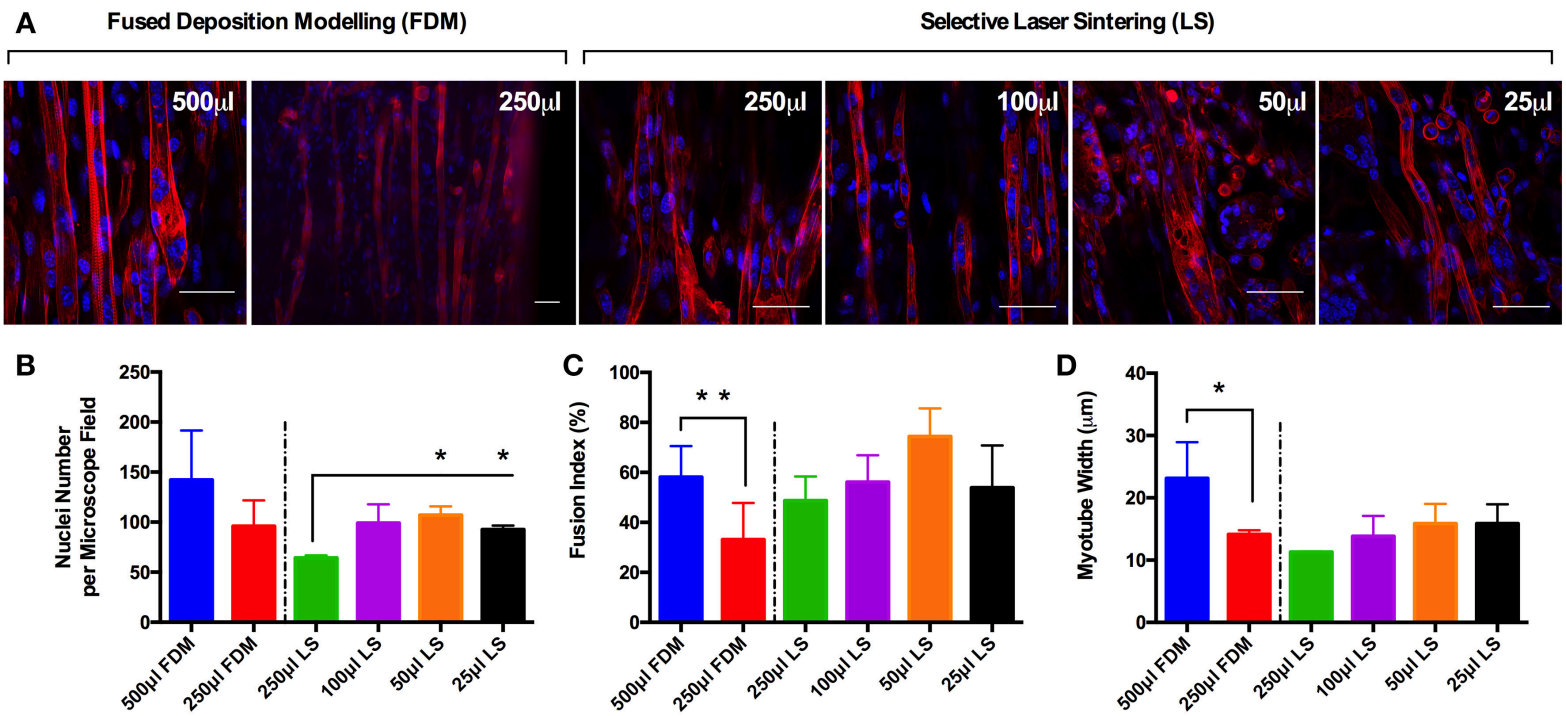
**(A)** Morphological staining of the actin cytoskeleton (red) and nucleic DNA (blue) for each of the C_2_C_12_ muscle constructs after 14 days in culture. **(B)** Average nuclei number per microscope field for each of the C_2_C_12_ muscle constructs after 14 days in culture. **(C)** Fusion index for each of the C_2_C_12_ muscle constructs after 14 days in culture. **(D)** Average myotube width for each of the C_2_C_12_ muscle constructs after 14 days in culture. Data presented as mean ± SD from *n* ≥ 3 experimental repeats in each condition. ^*^P ≤ 0.05, ^**^P ≤ 0.01. Scale bar = 50 μm.

Observations of the morphology of each muscle constructs (Figure 2A) demonstrated no significant differences in nuclei number per microscope field for cells cultured in the 500 and 250 μL FDM constructs, as well as the 100, 50 and 25 μL (https://doi.org/10.17028/rd.lboro.6969683.v1) LS constructs. However, reduced nuclei numbers (Figure 2B) were observed in cells cultured in the 250 μL LS constructs when compared with the 50 (*P* ≤ 0.05) and 25 μL LS constructs (*P* ≤ 0.05). Variations in fusion index (Figure 2C) were observed when comparing between the 500 and 250 μL FDM constructs (*P* ≤ 0.01), however no significant differences were observed when comparing between any of the other conditions. Finally, variations in myotube widths (Figure 2D) were observed when comparing between the 500 and 250 μL FDM constructs (*P* ≤ 0.05), however again no significant differences in myotube width were evident between any other conditions. The combined morphological analyses suggest that whilst cells cultured in the FDM 250 μL inserts exhibited reduced myotube width and fusion index when compared with the FDM 500 μL inserts, each of the printed inserts irrespective of printing process or volume produced a morphologically viable, reproducible and scalable model.

### Reproducible Myotube Density and Length Within Tissue Engineered Muscle Models Between Construct Volumes of 500–50 μL

**Figure 3:**
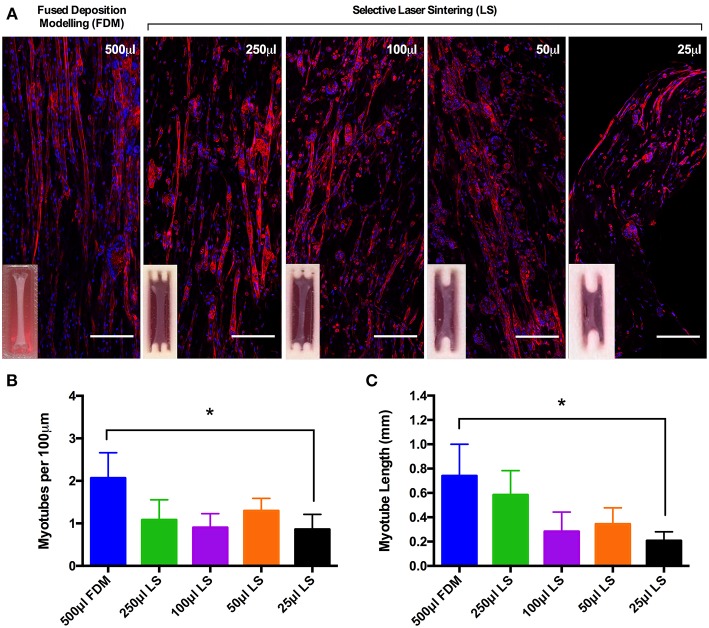
**(A)** Morphological staining of the actin cytoskeleton (red) and nucleic DNA (blue) for each of the constructs after 14 days in culture. Images captured via confocal tile scan (Zeiss LSM 880) and stitched together enabling visualization of the whole construct. **(B)** Myotube density within the collagen construct, expresses as myotubes per 100 μm, for each of the muscle constructs after 14 days in culture. **(C)** Average myotube length for each of the muscle constructs after 14 days in culture. Data presented as mean ± SD from *n* ≥ 3 experimental repeats in each condition. ^*^P ≤ 0.05. Scale bar = 100 μm.

Myotube density, expressed as myotubes per 100 μm of confocal tile scans (Figure 3A), was reproducible between collagen constructs of volumes from 500 and 50 μL, however statistical differences were observed between the largest (500 μL) and smallest (25 μL) construct volumes (*P* ≤ 0.05, Figure 3B). The same trend was observed when analyzing average myotube length within the constructs, with no observable statistical differences between any of the construct volumes from 500 and 50 μL. Reductions in average myotube lengths were however observed when comparing between the 500 and 25 μL constructs (*P* ≤ 0.05, Figure 3C). This data again indicates that these constructs enable the generation of reproducible and scalable collagen skeletal muscle constructs down to the size of 50 μL.

### Addition of Matrigel® Matrix to Tissue Engineered Muscle Models Elicits Increased Myotube Density and Myoblast Fusion

**Figure 4:**
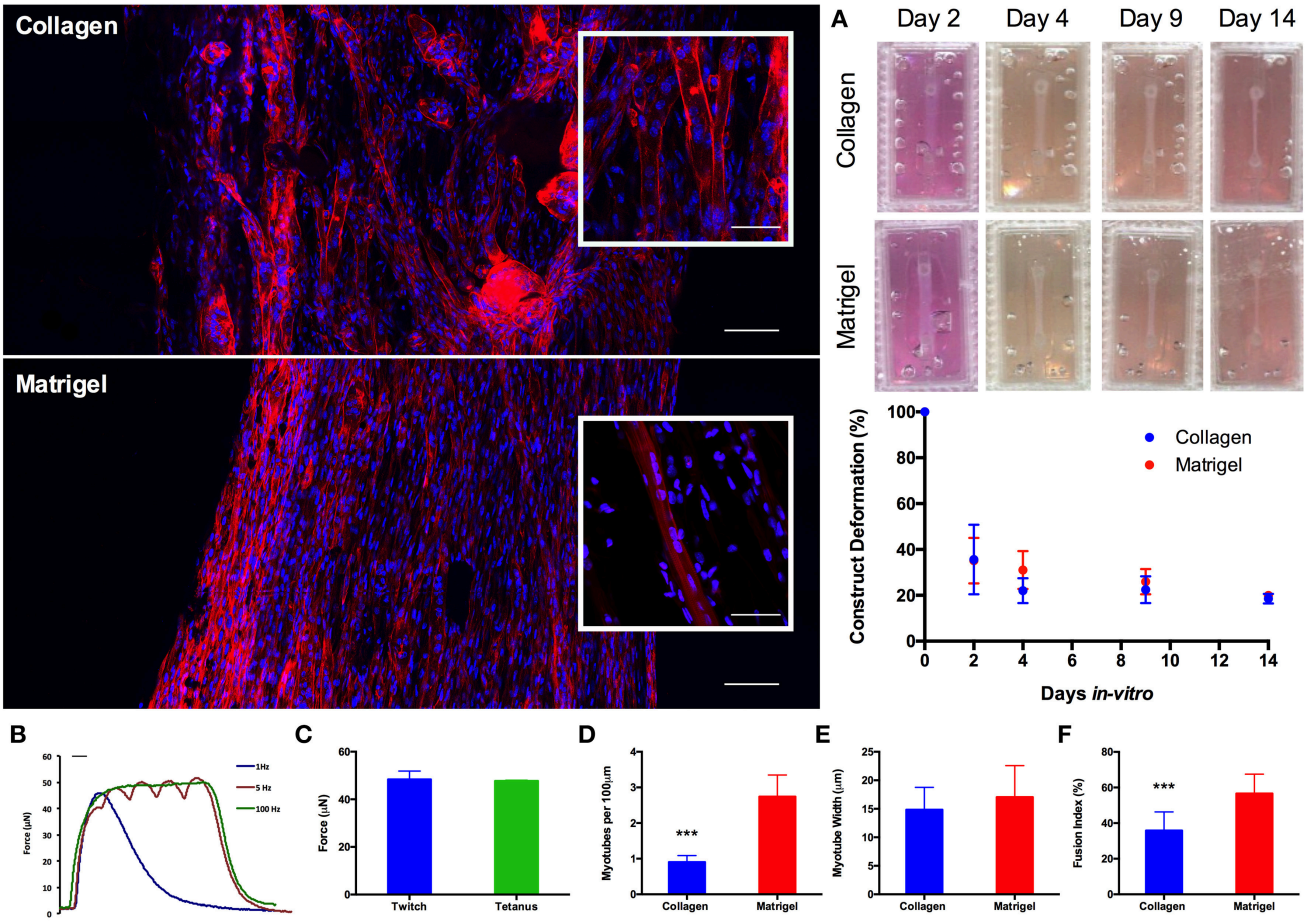
Morphological staining of the actin cytoskeleton (red) and nucleic DNA (blue) for collagen only and collagen/Matrigel® C_2_C_12_ muscle constructs after 14 days in culture. Images captured via confocal tile scan (Zeiss LSM 880) and stitched together enabling visualization of the whole construct. **(A)** Macroscopic images and construct deformation analysis of C_2_C_12_ muscle constructs after 2, 4, 9, and 14 days culture, for collagen only and collagen/Matrigel® constructs. **(B)** Contractile force measurements for collagen/Matrigel® C_2_C_12_ muscle constructs exposed to electrical field stimulation at 1, 5, and 100 Hz. **(C)** Average maximal twitch force and maximal tetanic force measurements for collagen/Matrigel® C_2_C_12_ muscle constructs exposed to electrical field stimulation. **(D)** Myotube density within the collagen only and collagen/Matrigel® C_2_C_12_ muscle constructs, expressed as myotubes per 100 μm, for each constructs after 14 days in culture. **(E)** Average myotube width for each of the C_2_C_12_ muscle constructs after 14 days in culture. **(F)** Myotube fusion index for each of the C_2_C_12_ muscle constructs after 14 days in culture. Data presented as mean ± SD from *n* ≥ 3 experimental repeats in each condition. ^***^P ≤ 0.001. Scale bar = 100 μm for main body stained images, 50 μm for inset stained images, 100 ms for **B**.

Having established scalability within the model, 50 μL constructs (https://doi.org/10.17028/rd.lboro.6969710.v1) were selected as a suitable construct size and cell seeding number (2 × 10^5^ cells per construct). Whilst LS has been demonstrated as a viable manufacturing option, reduced print material cost (~£30 vs. ~£200 per kg), machine cost (<£1 k vs. >£100 k) and consequently availability, as well as the necessity to post-process LS parts to remove non-sintered PA-12 particles from the molds, all made FDM the more favorable print process for this application. As such, molds were re-designed to incorporate a removable chamber (https://doi.org/10.17028/rd.lboro.6969707.v1, Figure S1) preventing any unwanted adhesion between the matrix and the molds; making FDM a viable manufacturing process at the required scale. Furthermore, the inclusion of Matrigel®; containing basement membrane proteins, into the model increased the biological relevance of the hydrogel composition. *In vivo* muscle fibers are encased within a basement membrane containing a range of pericellular proteins. This close association of sarcolemma and basement membrane creates an important biological niche in which satellite cells, the stem cells of muscle, reside. Morphological comparisons between C_2_C_12_ muscle constructs demonstrated significant (*P* ≤ 0.001) increases in myotube density (expressed as myotubes per 100 μm) for constructs containing Matrigel® matrix (65% type 1 rat tail collagen, 20% Matrigel®), when compared to constructs manufactured from only collagen (85% type 1 rat tail collagen). Likewise, constructs containing Matrigel® demonstrated increased myoblast fusion (*P* ≤ 0.0005) when compared with the collagen only gels. No significant differences were observed between the two construct types when assessing average myotube widths (*P* ≥ 0.05). Reproducible construct deformation was observed across construct types indicating homogeneous C_2_C_12_ re-modeling of the extracellular matrix regardless of matrix composition. Muscle function within the Matrigel® constructs was assessed through the production of contractile force when exposed to electrical field stimulation at 1, 5, and 100 Hz (Figure 4), exhibiting significantly enhanced force compared to collagen only matrices. This stimulation generated an average maximal twitch force of 48.39 ± 3.49 μN, as well as an average maximal tetanic force of 47.74 ± 0.31 μN. Force generated signifies an increase in construct maturation compared to collagen only hydrogels at both 65 (4.87, 6.02 μN) and 85% (4.16, 5.98 μN) matrix composition for twitch and tetanus contractions respectively (Figure S2).

### Functional Tissue Engineered Human Derived Skeletal Muscle Constructs (50 μL, 2 × 10^5^ Cells Per Construct)

**Figure 5:**
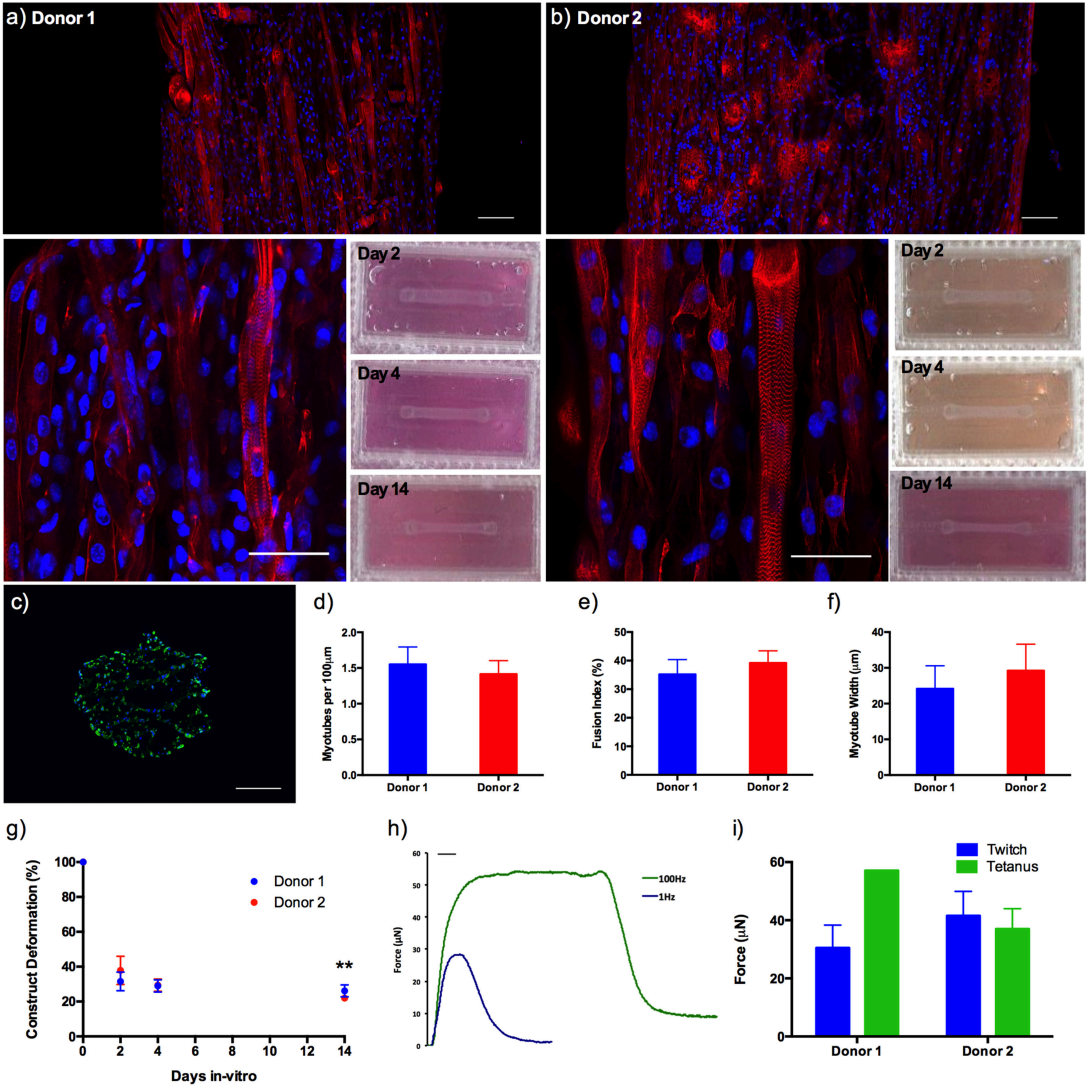
**(a,b)** Morphological staining of the actin cytoskeleton (red) and nucleic DNA (blue) for human derived muscle cell constructs from donor 1 and donor 2 after 14 days in culture, as well as macroscopic images of construct deformation over 2, 4, 9, and 14 days culture. Images captured via confocal tile scan (Zeiss LSM 880) and stitched enabling visualization of the whole construct. **(c)** Morphological staining of the muscle specific protein filament Myosin Heavy Chain (green) and nucleic DNA (blue) on histological cross-section. **(d)** Myotube density within the collagen/Matrigel® human derived muscle cell constructs, expressed as myotubes per 100 μm, for each constructs after 14 days in culture. **(e)** Myotube fusion index within the collagen/Matrigel® human derived muscle cell constructs, expressed as myotubes per 100 μm, for each constructs after 14 days in culture. **(f)** Average myotube length within the collagen/Matrigel® human derived muscle cell constructs, expressed as myotubes per 100 μm, for each constructs after 14 days in culture. **(g)** Construct deformation analysis of collagen/Matrigel® human derived muscle cell constructs after 2, 4, 9, and 14 days culture. **(h)** Representative tetanic and twitch contractile force measurements for human derived muscle cell constructs. **(i)** Average maximal twitch force and maximal tetanic force measurements for human derived muscle cell constructs exposed to electrical field stimulation. Data presented as mean ± SD from n ≥ 3 experimental repeats in each condition. ^**^P ≤ 0.01. Scale bar = 100 μm for tile scan images (top), 50 μm for individual stained images (middle), 100 ms for **f**.

Morphological analysis between donors demonstrated no significant variations in average myotube density (expressed as myotubes per 100 μm), fusion index or average myotube length (all *P* ≥ 0.05), representing a reproducible morphological response within the model regardless of the HDMC source. Likewise, construct deformation for both donors was reproducible, indicating homogeneous remodeling of the extracellular matrix. Muscle function within the HDMC constructs was assessed through the production of contractile force when exposed to electrical field stimulation at 1 and 100 Hz. This stimulation generated an average maximal twitch force of 30.45 ± 7.86 μN for donor 1 and 41.52 ± 8.38 μN for donor 2, as well as an average maximal tetanic force of 57.07μN for donor 1 and 36.96 ± 7.04 μN for donor 2 (Figure 5).

## Discussion

Establishing a model that closely represents native *in vivo* skeletal muscle is of paramount importance if such *in vitro* systems are to have utility in understanding the physiology of skeletal muscle development, as well as the underpinning mechanisms associated with health and disease within this tissue. Furthermore, it is essential that where appropriate primary human tissue is utilized, due to the issues associated with accurate translation of *in vitro* cell line and *in vivo* animal data to human clinical trials. However, obtaining large numbers of myogenic cells from human biopsies is experimentally laborious, time consuming and presents challenges associated with maintaining cellular phenotype across serial passages. Attempts have been made to extend the number of passages tolerated by primary skeletal muscle cells (Penton et al., 2016), however, when using primary human cells reducing the number of cells required per experiment is still paramount to increasing throughput. Other potential applications of tissue engineered muscle, including transplantation of large tissue volumes, may necessitate the use of constructs of varying size and cell number, and as such for these models to have a significant impact in the basic and applied sciences, it is essential they are time and cost efficient to produce, facilitate scalability and allow flexibility of application. Here we have presented a rapid, reliable and scalable model which produces TE human skeletal muscle within a modified type-1 collagen hydrogel that closely represents the structure and function of native *in vivo* tissue. The model presented in this research provides the first viable and morphologically mature TE primary skeletal muscle model, generated using commercially available 3D printing and open access designs, allowing accurate translation of results between laboratories. The scaling work presented (≥1 × 10^5^ cells per construct) facilitates the generation of multiple constructs per donor biopsy enhancing experimental throughput.

Type-1 collagen is present in the epi-, peri-, and endomysial layers of skeletal muscle (Kjaer, 2004), with this extracellular protein being extensively used in the generation of hydrogel-based skeletal muscle that is highly organized in nature (Cheema et al., 2003; Vandenburgh, 2010; Hinds et al., 2011). These systems are considered more favorable than conventional monolayer cultures of myotubes (early pre-mature muscle fibers), owing to the *in vivo*-like alignment, the enhanced experimental longevity and the organization of myotubes in three dimensions (Vandenburgh et al., 1988). Most models share the same basic principle of hydrogel attachment to anchor materials that act as pseudo-tendons, to provide the mechanical signal required for cell alignment and differentiation. This has previously been achieved through the use of multiple materials including; polyethylene meshwork (Brady et al., 2008; Mudera et al., 2010; Player et al., 2014), Velcro® tabs (Boonen et al., 2010; Langelaan et al., 2011) and polydimethylsiloxane (PDMS) posts (Vandenburgh, 2010). Advancements in additive manufacture (AM) have prompted a trend toward TE platforms (Truskey et al., 2013) that are manufactured by automated technology (Costa et al., 2015; Zhang and Zhang, 2015), due to the associated design flexibility of processes, such as 3D printing. The model presented here, demonstrates the use of biocompatible PLA and PA-12 (Rimington et al., 2017, 2018) molds building on previously published molds that feature a twin or single based post design at decreasing construct volumes, utilizing user-friendly accessible FDM and LS 3D printing techniques (Jones et al., 2018). Such designs ensure there is minimal friction between the side surfaces of the constructs; eliciting cell-mediated mechanical matrix contraction in the X axis that facilitates myoblast alignment and myogenic differentiation for the formation of 3D multinucleated myotubes.

Deformation analysis of scaled constructs appeared to indicate favorable matrix contraction of FDM 500 μL hydrogels across early time-points. However, such discrepancies were largely negated after 14 days in culture in scaled FDM and LS constructs; indicating comparable cellular attachment and mechanical contraction independent of polymer or construct volume. Such responses may allude to a non-significant effect of polymer/matrix interaction when scaling 3D printed collagen gel constructs. It was observed that complete matrix detachment from the mold material surrounding the construct posts facilitated complete matrix deformation, ensuring uniaxial cell-mediated matrix contraction in the Y axis and preventing conflicting strain in the X axis. The probability of incomplete matrix detachment appeared to increase with reducing construct volume, necessitating the development of the two-part construct design with a removable barrier (Figures S1E,I), ensuring complete matrix detachment from the mold after 48 h (Figure 4A).

Comparisons of morphological measures (nuclei number, myotube fusion index, myotube width and length, and myotube density) across each of the scaled volumes were highly reproducible. Although reduced fusion efficiency and myotube widths were observed within FDM 250 μL constructs, hydrogels still evidenced viable morphological development. In addition, reductions in this scaled range were offset by comparable myotube fusion index and width observed in LS 250 μL constructs. When assessing all morphological measures as one body of evidence the experimental procedure utilized provides a robust and reproducible model, irrespective of manufacturing process or construct volume. The manufacturing process of choice is therefore a selection between the inexpensive, disposable FDM molds or the more expensive but autoclavable PA-12 LS molds. Further examination of entire scaled collagen constructs (FDM; 500 μL, LS; 250–25 μL), utilizing confocal tile stitching to create “macroscopic” images of microscopic cellular regions (Figure 3) further outlined effective, reproducible scaling of *in vivo*-like skeletal muscle. Differences in length, and density of myotube distribution were evident in LS 25 μL constructs compared to FDM 500 μL. However, such differences may be a consequence of the advanced biomimetic environment observed within FDM 500 μL constructs; entailing densely packed aligned myotubes of up to 1 mm in length with abundant sarcomeric organization. As such, although it is reasonable to suggest that viable cultures of multinucleated myotubes remained evident within this scaled volume, it is recommended that scaling within the current model be refined to the range of 500–50 μL volumes. To further assist AM technique selection to replicate designs within this work, a comprehensive summary figure detailing the steps of manufacture and their specific advantages for tissue engineering applications is provided in the supplementary information section (Table S2).

Addition of 20% v/v Matrigel® matrix to TE C_2_C_12_ muscle models was shown to significantly increase morphological (myotube density and fusion index) and functional (force output, Figure S2) measures of tissue maturation, whilst also maintaining uniform cell-mediated matrix remodeling as in the collagen only constructs (Figure 4). Specifically, collagen-Matrigel® constructs were shown to exhibit a 3-fold increase in myotubes per 100 μm (2.74 ± 0.71 vs. 0.90 ± 0.18), as well as substantially increased fusion index (56.62 ± 10.86 vs. 35.81 ± 10.46%) when compared to the collagen only models. Both average myotube width and construct deformation were consistent across both conditions. The scalability of the model (>1 × 10^5^ cells per model) would also enable primary HDMC constructs to be realized in quantities suitable for substantial experimental and biological repeats from each muscle donor (up to 500). These HDMC constructs demonstrated the same abundance of aligned multinucleated myotubes as the C_2_C_12_ cell line models, as well as exhibiting muscular striation indicative of mature tissue (Figure 5). This maturation was confirmed morphologically with average myotube widths in excess of 24 μm, myotube densities in excess of 1.4 myotubes per 100 μm, as well as fusion index values in excess of 35% for both donors. Functional contractile force measurements in response to electrical field stimulation generated twitch force in excess of 30 μN (1 Hz), as well as maximal tetanic forces in excess of 36 μN (100 Hz) for both donors. The tetanic force traces generated also demonstrated the capacity of these constructs to contract and relax in response to electrical excitation, mimicking the behavior of *in vivo* tissue (Figure 5). In addition to the generation of highly mature primary human muscle tissue, the addition of Matrigel® matrix supplemented the model with basement membrane proteins, increasing biomimicry within the system. This has been previously reported in fibrinogen/Matrigel® constructs, in which additive addition of the basement membrane component resulted in enhanced tissue maturation (Hinds et al., 2011). The precise composition of these matrix components can have a profound effect upon the biophysical and biochemical interactions that regulate biological behavior within the tissue. For example, it is known that matrix elasticity and hydrogel stiffness (elastic modulus) within both synthetic and naturally derived polymer scaffolds can direct stem cell differentiation (Engler et al., 2006), regulate cellular migration, spreading and adhesion (Pelham and Wang, 1997; Zaman et al., 2006; Olsen et al., 2011) and specifically within tissue engineered muscle will modulate force production (Hinds et al., 2011). Collagen-1 hydrogels of concentration 1, 3, and 7mg/mL have been recorded with elastic modulus of 5.0 ± 0.6, 55.4 ± 11.0, and 341.8 ± 32.4 Pa (Slater et al., 2017). Materials characterization of high collagen (4mg/mL), collagen/Matrigel® (2/2 mg/mL) and collagen/ high Matrigel® (2/4 mg/mL) compositions has also been documented (Anguiano et al., 2017) with increases in fiber diameter and pore size observed with the addition of Matrigel®. It should be noted that Matrigel® supplement is an animal derived basement membrane extract (secreted by Engelbreth-Holm-Swarm mouse sarcoma cells), and as such may exhibit variability between commercial suppliers and batches. As such it is critical that experimentation using this supplement is highly controlled, with controls and experimental conditions derived from single batches to ensure variability does not confound results.

Precision scaling of bioengineered skeletal muscle presented within this work across a range of 500–50 μL constructs, allows for the generation of both cell line and primary tissues that can be used in high content experimental screening prior to larger scale *in vitro* and small animal *in vivo* studies (Vandenburgh, 2010). Moreover, it is possible to generate multiple analytical; molecular (gene expression), morphological (histology) and functional (force generation) data from the presented constructs, demonstrating its diverse utility during biological investigations. The scalability of the model presented makes it highly suited to using primary MPC derived from multiple species. This work builds on the previous human TE models previously produced (Powell et al., 2002; Mudera et al., 2010; Martin et al., 2013; Madden et al., 2015), however significantly reduces the cell seeding density required to produce densely packed and functionally mature tissues. Previous models utilizing sutra pins and fibrinogen matrixes have reported seeding numbers as low as 1 × 10^5^, however viable tissue was only established at 4 × 10^5^ densities (Martin et al., 2013). Bespoke systems using fibrinogen (Madden et al., 2015) and type-1 collagen (Powell et al., 2002), both combined with Matrigel® required seeding densities of 7.5 × 10^5^ and 1.2 × 10^6^ cells per mold respectively. The seeding density for scaled constructs reported in this work would elicit a 50% decrease in the cell number required to produce mature tissue, when compared to the lowest viable density previously reported. Furthermore, the bespoke nature of many of the previously published systems elicits issues in experimental scale up. This significantly enhances the amount of tissue producible from each donor biopsy, while also satisfying the 3R's principles of biological research (Replacement, Refinement, and Reduction). Should primary animal cells be used, the models presented here enhance the number of constructs and experiments (repeated measures) that can be conducted using tissue isolated from a single donor animal.

Current and future work surrounding skeletal muscle will focus on the successful integration of supporting cells types. Here, the ability to design and, crucially, adapt biocompatible 3D printed constructs to facilitate the development of the desired biological microenvironment will be of paramount importance. As such, the adoption of methods that incorporate the flexibility and rapid prototyping capability of 3D printing demonstrated within this work will contribute significantly to the advancement of skeletal muscle and provide concepts which can apply to other engineered tissues in the future.

## Materials and Methods

### Design and Fabrication of 3D Printed Well-Insert Molds

3D printing was performed either by FDM or LS. Molds were designed with a single or twin post fixed at the end of a rectangular mold (Figure S1). Mold and post dimensions were scaled to match the volume of collagen required for each specific construct (Table S1). 50 μL constructs were assembled in 2-parts with a removable barrier. The external geometries of the part were designed to fit into a standard 6 or 12 well culture plate. All 3D modeling was performed using computer aided design (CAD) Siemens NX software (version 8.5); with completed.stl files verified using Materialize MiniMagics. FDM printing utilized a commercially available Ultimaker 2+ system (Ultimaker, Netherlands). For FDM, completed.stl files were processed using the in-house Cura Software for Ultimaker 2+ (version 3.2). FDM parts were printed using PLA and were extruded onto the standard glass build plate, at previously published settings (Rimington et al., 2017). LS parts were printed using an EOS Formiga P100™ (EOS GmbH, Germany) from PA-12. The powder used was a mixture of recycled and virgin powder (20% recycled, 80% virgin); well within manufacturer recommendations. Samples were removed from the build chamber and cleaned using a soft abrasive brush to remove un-sintered powder. All samples were sterilized via UV for ≥1 h, prior to being adhered to culture well plates using an in-house bio-adhesive which has been found to be completely compatible (Rimington et al., 2017). Once adhered, samples were rinsed with 70% IMS and left for the remaining solvent to evaporate prior to use.

### Cell Culture

#### Culture of C_2_C_12_ Skeletal Muscle Myoblast Cells

C_2_C_12_ skeletal muscle myoblast cells (ECACC, all below passage 10) were grown using standard growth medium (GM); composed of Dulbecco's Modified Eagle's Medium (DMEM, Fisher–Scientific, UK), 20% fetal bovine serum (FBS, Pan Biotech, UK), and, 1% Penicillin/Steptomyocin (P/S, Fisher–Scientific, UK). Cells were cultured in T80 flasks (Nunc™, Fisher–Scientific, UK) and incubated in a 5% CO_2_ humidified atmosphere at 37°C until 80% confluence was attained. GM was changed every 24 h during the culture period for expansion of cells.

#### Isolation and Subsequent Culture of Human Derived Muscle Cells (HDMCs)

Healthy males volunteered for this study, which was approved by the Loughborough University Ethics Approvals (Human Participants) Sub Committee (reference number: H14-P16). Before participation, subjects provided written informed consent and completed a medical screening questionnaire. HDMC samples were obtained from healthy males (*n* = 2), between the ages of 18–55 reporting no recent injuries or intake of anti-inflammatory pharmaceuticals. Tissue was obtained via the Bergstrom biopsy procedure, with any visible connective tissue being removed (Bergstrom, 1975). Tissue samples were removed from the storage GM solution and washed three times in a buffer solution (PBS, 1% P/S & 1% Amphotericin, Sigma, UK). Once washed tissue chunks were placed into a petri dish, suspended in 1 mL of GM and mechanically minced using 2 scalpel blades until broken down into small sized pieces. Tissue was then seeded into 0.2% gelatin (Sigma, UK) coated T25 flasks (approximately 4 pieces/flask) and suspended in 0.5 mL of GM to ensure tissue was planted on the culture surface and not floating. Flasks were then placed in standard tissue culture incubators (37°C humidified atmosphere/5% CO_2_) for 7–10 days to allow cellular migration to occur with more GM added to prevent flasks drying out. Migration of HDMC's was monitored with the migrated cellular population passaged at 60% confluence to prevent spontaneous differentiation at low passage. Cells were dissociated with accutase (Fisher-Scientific, UK) before re-plating. HDMC's were then cultured through three passages to confirm myogenic capacity and increase cell quantity for experimental purposes. All HDMC's were used between passages 3 and 6 as previously published by our group (Martin et al., 2013).

### Tissue Engineered Constructs

**Figure 6:**
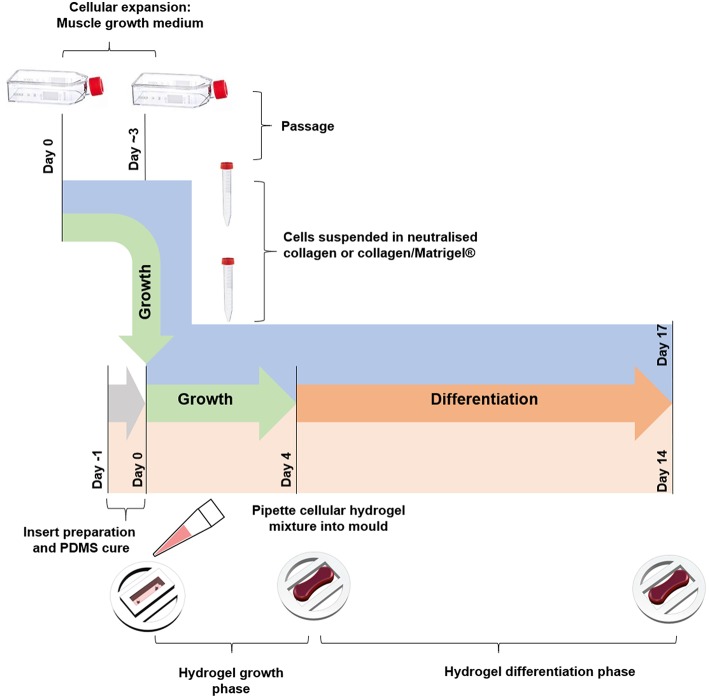
Schematic diagram for tissue engineered skeletal muscle fabrication.

Collagen constructs were generated using C_2_C_12_ myoblasts, with the method based around previous work from our group (Mudera et al., 2010). Collagen hydrogels were formed by the addition of 85% v/v type I rat tail collagen (First Link, UK; dissolved in 0.1 M acetic acid, protein at 2.035 mg per mL), with 10% v/v of 10X minimal essential medium (MEM) (Gibco, UK). This solution was subsequently neutralized by the addition of 5 and 1 M sodium hydroxide (NaOH) dropwise, until a color change to cirrus pink was observed. The cells were added at a seeding density of 4 × 10^6^ cells per mL in a 5% v/v GM solution, before being transferred to the pre-sterilized inserts (various construct sizes, Figure 1) to set for 10–15 min at 37 °C. GM was added for 4 days and changed daily, before being changed to differentiation media (DM, DMEM, 2% Horse Serum (HS), 1% P/S) for a further 10 days in culture. Collagen/Matrigel® constructs were generated using both C_2_C_12_ myoblasts (Figure 4) and HDMCs (Figure 5) in removable 50 μL molds (Figure S1) 3D printed via FDM. Gels were formed by the addition of 65% v/v type I rat tail collagen, with 10% v/v of 10X minimal essential medium (MEM) (Gibco, UK). This solution was subsequently neutralized by the addition of 5 and 1 M sodium hydroxide (NaOH) dropwise, until a color change to cirrus pink was observed. This was followed by the addition of 20% v/v Corning® Matrigel® Matrix (Corning, Germany). The cells were added at a seeding density of 4 × 10^6^ cells per mL in a 5% v/v GM solution, before being transferred to the pre-sterilized inserts to set for 10–15 min in an incubator. GM was added for 4 days and changed daily, before being changed to DM for a further 10 days in culture. Figure 6 outlines the process for fabricating tissue engineered skeletal muscle.

### Cryosectioning

Fixed constructs were dehydrated in 10% (24 h) sucrose followed by 20% (24 h) sucrose (w/v in Tris-buffered saline (TBS). Constructs were then embedded in Tissue-Tek® (VWR, USA) optimum cutting temperature (O.C.T) mounting medium and frozen at −80°C for a minimum of 2 h. Once frozen 12 μm sections were prepared, using standard cryostat protocols, perpendicular to the longitudinal axis of the construct.

### Immunohistochemistry

Following the 14 days culture period, constructs were fixed using methanol and acetone at increasing concentrations. For C_2_C_12_ and HDMC constructs, the actin cytoskeleton was identified using Rhodamine-Phalloidin (1:500, Thermo-Fisher, UK) and nuclei were stained using DAPI (1:2,000, Thermo-Fisher, UK) in Tris-Buffered Saline (TBS) for 2 h. Slides were then washed (3 × 15 min, TBS) and transferred to poly-lysine microscope slides (Thermo-Fisher, UK) and mounted using Fluoromount™ mounting medium (Sigma-Aldrich, UK). For cryosections of HDMC constructs, sections were stained for Desmin to show myogenic cells and DAPI. Briefly sections were incubated overnight with anti-desmin antibody (1:200, Dako, UK, clone D33) in blocking solution (Tris-buffered saline mixed with polysorbate 20, TBST, 5% Goat serum). Samples were washed (3 × 15 min, TBS) and incubated for 1 h with secondary fluorescent antibody (1:500, Invitrogen, UK, Alexa Fluor™ 588 goat anti-mouse) and DAPI (1:2,000) in blocking solution. Slides were then washed (3 × 15 min, TBS) and mounted as previously described.

### Analytical Procedures

Measuring construct width provides an indication of cell attachment and remodeling and as such, images of the macroscopic contraction of the constructs were taken throughput the culture period using an EPSON flatbed scanner [Perfection V330 Photo, 300DPI, (Smith et al., 2012)]. Construct width was measured using FIJI image analysis software [version 1.5a, (Schindelin et al., 2012)]. Images were captured using a Leica DM2500 or a Zeiss LSM 880 confocal and used to analyse the morphology of the myotubes including; fusion efficiency (number of nuclei in myotubes represented as a percentage of the total number of nuclei in the image frame), myotube density per 100 μm (number of myotubes measured across a 100 μm cross section of the gel), myotube length and myotube width. Analysis was conducted from 5 images taken from each construct, within each condition and repeated at least three times (*n* ≥ 3).

### Assessment of Muscle Function by Electrical Stimulation

Constructs were washed twice in PBS and one end of the construct was removed from the mold. The loose end of the construct was then attached to the force transducer (403A Aurora force transducer, Aurora Scientific, UK) using the eyelet present in the construct. The construct was covered (3 mL) with Krebs-Ringer-HEPES buffer solution (KRH; 10 mM HEPES, 138 mM NaCl, 4.7 mM KCl, 1.25 mM CaCl_2_, 1.25 mM MgSO, 5 mM Glucose, 0.05% Bovine Serum Albumin in dH_2_0, Sigma, UK). Wire electrodes were positioned either side of the construct to allow for electric field stimulation. Impulses were generated using LabVIEW software (National Instruments, Berkshire, United Kingdom) connected to a custom-built amplifier. Maximal twitch force was determined using a single 3.6 v/mm, 1.2 ms impulse and maximal tetanic force was measured using a 1 s pulse train at 100 Hz and 3.6 v/mm, generated using LabVIEW 2012 software (National Instruments, UK). Where possible, twitch and tetanus data were derived from three contractions per construct. Data was acquired using a Powerlab system (ver. 8/35) and associated software (Labchart 8, AD Instruments, UK).

### Statistical Analysis

Significance of data were determined using IBM^©^ SPSS^©^ Statistics version 23. Mauchly's test of sphericity and Shapiro-Wilk tests were used to confirm homogeneity of variance and normal distribution of data, respectively. Where parametric assumptions were met, a 4 × 6 (Figure 1), 2 × 5 (Figure 4), or 2 × 4 (Figure 5) factorial analysis of variance (ANOVA) was used for construct deformation analyses. One-way ANOVA (1 × 4) was used to analyse morphological data; myotube number, myotube width, fusion index, nuclei number, myotube density, and myotube length only concerned with experimental termination time-points. Where significant interactions were observed, Bonferroni *post-hoc* analyses were used to analyse differences between conditions at specific time-points. Non-parametric Kruskal-Wallis (H) analysis was undertaken where data violated parametric assumptions. Mann-Whitney (U) tests were then utilized to determine significance between conditions, in accordance with Bonferroni correction to account for incremental type-1 error. All data is reported as mean ± standard deviation (SD). Significance was assumed at *P* ≤ 0.05.

## List of Available Designs Used Within This Work

500 μL Mold FDM—Available online at: https://doi.org/10.17028/rd.lboro.6969851.v1.

250 μL Mold FDM and LS—Available online at: https://doi.org/10.17028/rd.lboro.6969848.v1.

100 μL Mold LS—Available online at: https://doi.org/10.17028/rd.lboro.6969806.v1.

50 μL Mold LS—Available online at: https://doi.org/10.17028/rd.lboro.6969797.v1.

50 μL Mold FDM—Available online at: https://doi.org/10.17028/rd.lboro.6969710.v1.

50 μL FDM removable insert—Available online at: https://doi.org/10.17028/rd.lboro.6969707.v1.

25 μL Mold LS—Available online at: https://doi.org/10.17028/rd.lboro.6969683.v1.

## Author Contributions

Design and 3D printing were conducted by AC and RR. Primary human biopsies were conducted by RF. Primary human tissue explant was conducted by NM. AC, RR, JF, DP, NM, JJ, and MT designed and conducted biological experiments. AC, RR, JF, MT, and LB contributed to analytical work. AC, RR, JF, and DP wrote the manuscript. VM and ML conceived the concept and reviewed the manuscript.

### Conflict of Interest Statement

The authors declare that the research was conducted in the absence of any commercial or financial relationships that could be construed as a potential conflict of interest.
