# Supplementary material for: Scalable 3D Printed Molds for Human Tissue Engineered Skeletal Muscle
Source: Front Bioeng Biotechnol. 2019 Feb 14;7:20. doi: 10.3389/fbioe.2019.00020 (PMC6383409; doi:10.3389/fbioe.2019.00020)
Supplement: Supplementary file 1 [file Data_Sheet_1.PDF]

Supporting Information

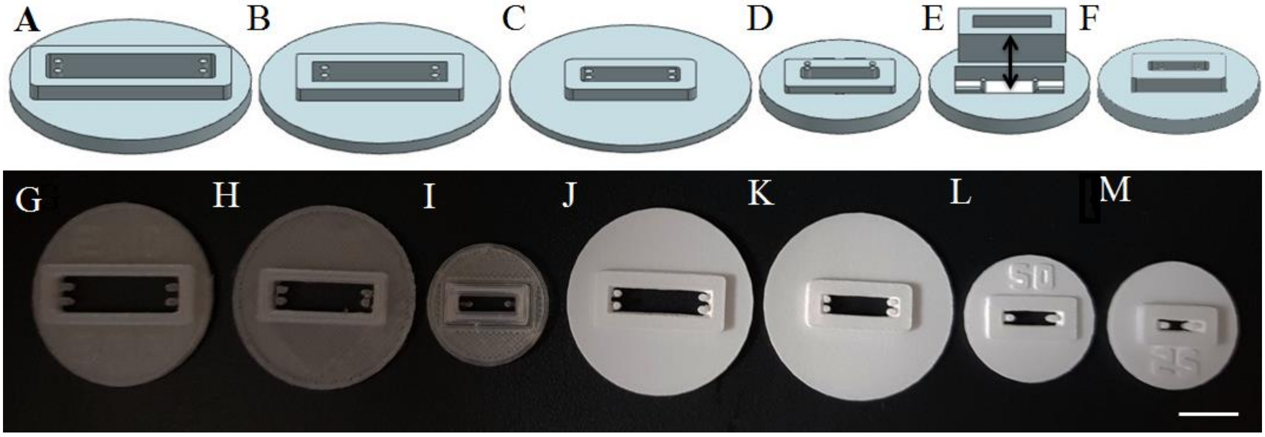

Fig.S1. CAD model of (A) 500 µL mould (B) 250 µL mould (C) 100 µL mould (D) 50 µL mould (E) 2-part 50 µL mould (F) 25 µL mould (G) 500 µL mould printed via FDM (H) 250 µL mould printed via FDM (I) 2-part 50 µL mould printed via FDM (J) 250 µL mould printed via LS (K) 100 µL mould printed via LS (L) 50 µL mould printed via LS (M) 25 µL mould printed via LS. Scale bar = 10 mm.

a)

	Matrigel	65 % Collagen	85% Collagen
	Volume per 100µl of hydrogel (µl)		
Acidic Collagen	65	65	85
EMEM	10	10	10
Matrigel™	20	-	-
Cells (4 x 10^{5}) in growth media	5	25	5

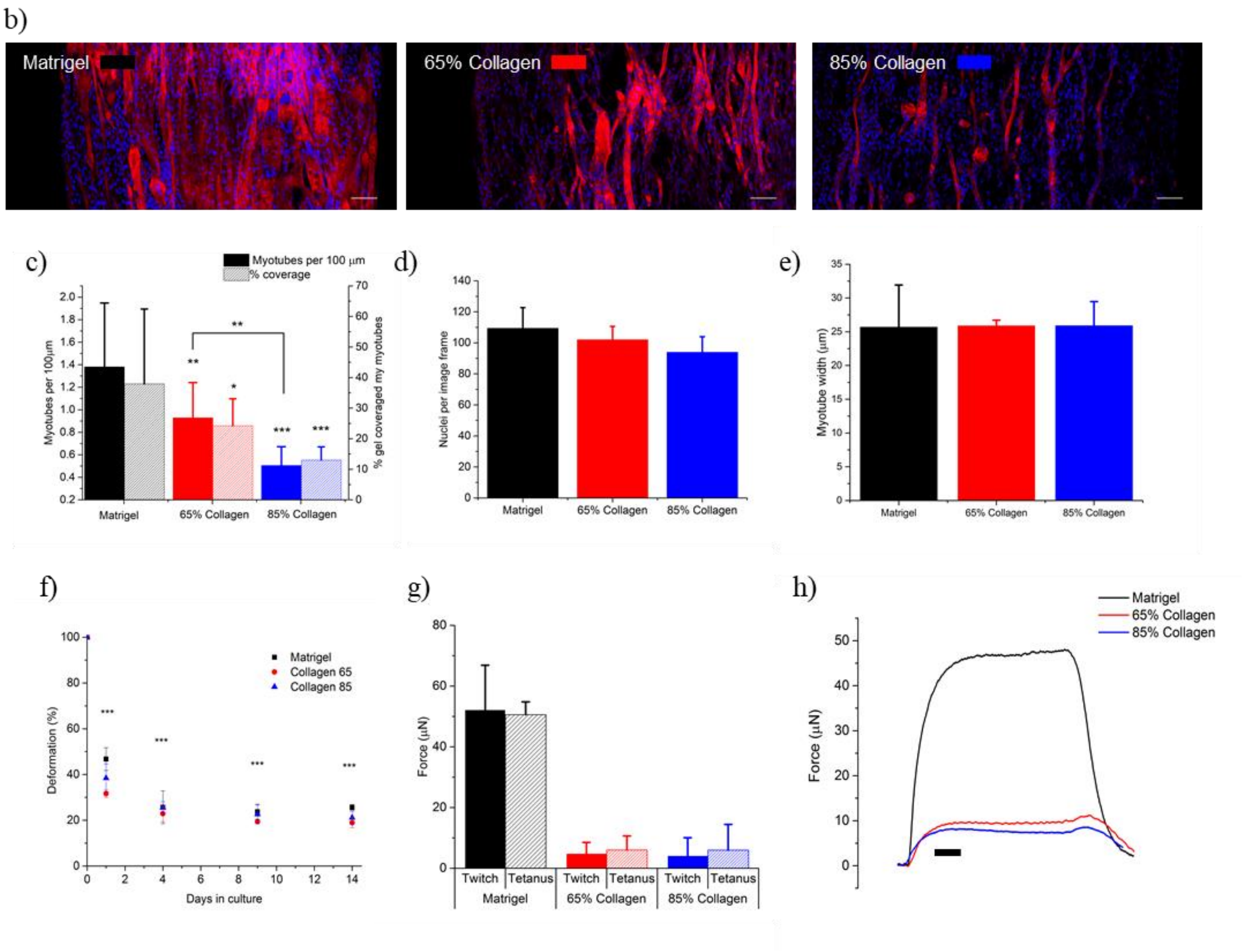

Fig.S2. Inclusion of Matrigel® into hydrogel constructs causes an increase in morphological maturity and force generation. (a) Table showing the composition of each hydrogel, volumes in table are per 100µl (two gels) and so can also be considered as percentages (b) Representative confocal micrographs at 40x magnification, phalloidin staining (red) and DAPI (blue) identify actin and nuclei respectively. Scale bars represent 100um (c) Myotube density expressed as myotubes per 100um (solid bars) and as percentage of gel covered by myotubes (hashed bars). Mean ± SD. Asterisks denote significance from control * $p<0.05$, ** $p<0.01$, *** $p<0.001$. (d) Nuclei per image frame Mean ± SD. (e) Myotube width, Mean ± SD. (f) Deformation calculated from images of hydrogels over time, expressed as a percentage of the area of the hydrogel at day 0, asterisks denote significance from control *** $p<0.001$. No significance between groups is present. (g) Twitch (1Hz) and Tetanus (100Hz) force measurements for 3 different hydrogel compositions. (h) Representative force traces for tetanic twitches for each hydrogel formulation. Scale bar represents 100ms.

Table S1: Internal culture dimensions for each of the collagen gel moulds used.

Collagen Gel Volume (μL)	Collagen Gel Length (mm)	Collagen Gel Width (mm)	Length:Width Ratio
500	21	6	3.5
250	16.7	4.8	3.5
100	12.3	3.5	3.5
50	9.7	2.8	3.5
25	7.6	2.2	3.5

Table S2: Fused Deposition Modelling (FDM) and Selective Laser Sintering (LS) technical specifications, manufacturing processes and applications for the proposed chambers.

	**Fused Deposition Modelling (FDM)**	**Laser Sintering (LS)**
**Equipment**	Ultimaker 2+	EOS Formiga P100
**Material Type**	Polylactic acid (PLA)	Polyamide-12 (PA-12)
**Print Resolution**	Nozzle size: 250 µm<br>Layer thickness: 100µm	Layer thickness: 60 µm
**Print/Equipment Cost**	Low	High
**Post Processing**	Not required	Un-sintered powder removal
**Commercial Availability**	High	Moderate
**Material Biocompatibility**	Excellent	Excellent
**Reusability**	Sterilisable via UV irradiation/alcohol immersion	Fully autoclavable
**Collagen Mould Scalability**	50 µL to 500 µL	50 µL to 500 µL
**Success Rate (%)**	> 90	>90
